# Social media harassment and psychological distress among collegiate student-athletes: a moderated mediation model of psychological resilience and perceived social support

**DOI:** 10.3389/fpsyg.2026.1885803

**Published:** 2026-10-01

**Authors:** Diyang Sun, Chan Lin, Caizhu Gao, Guoliang Li

**Affiliations:** 1 College of Physical Education, Kookmin University, Seongbuk-gu, Seoul, Republic of Korea; 2 College of Physical Education, Chizhou University, Chizhou, Anhui, China; 3 College of Physical Education, Anshan Normal University, Anshan, Liaoning, China

**Keywords:** collegiate student-athletes, digital stressor, moderated mediation, perceived social support, psychological distress, psychological resilience, social media harassment, sport psychology

## Abstract

**Introduction:**

Social media harassment is a sport-relevant digital stressor that may be associated with psychological distress among collegiate student-athletes. This study examined whether psychological resilience statistically accounted for part of this association and whether perceived social support showed both direct protective associations and a buffering role.

**Methods:**

A cross-sectional multi-site convenience sample of 426 collegiate student-athletes from university sport programs in China, the Republic of Korea, and the United States completed measures of social media harassment, psychological resilience, perceived social support, and psychological distress. Analyses included confirmatory factor analysis, country and gender comparisons, hierarchical regression, bootstrap mediation, conditional process analysis, and robustness checks.

**Results:**

The four-factor measurement model showed good fit and the focal scales showed acceptable reliability and convergent validity. Social media harassment was positively associated with psychological distress and negatively associated with psychological resilience. Perceived social support was associated with higher resilience, lower distress, and lower reported harassment. The indirect association through resilience was 0.133 (95% CI [0.088, 0.185]). Perceived social support moderated the harassment-resilience association (interaction = 0.12, *p* = 0.003). Conditional indirect associations decreased from 0.151 at low support to 0.067 at high support, with an index of moderated mediation of −0.042 (95% CI [−0.078, −0.012]). Country- and gender-based differences were small, and the focal pattern remained stable in sensitivity analyses.

**Discussion:**

The findings support a conditional association model in which resilience is an explanatory psychological resource and perceived social support has complementary direct and buffering roles. Because the data are cross-sectional, the results should not be interpreted as evidence of temporal causality.

## 1 Introduction

Social media has become deeply embedded in the everyday life of collegiate student-athletes. It allows athletes to communicate with peers, maintain team identity, build public visibility, connect with supporters, and develop an athletic identity beyond the physical boundaries of campus and competition (Kavanagh et al., 2022; National Collegiate Athletic Association, 2026b). This visibility, however, also exposes student-athletes to hostile digital interactions. Student-athletes may receive demeaning comments after poor performance, humiliating messages about body image or personal life, aggressive criticism related to team outcomes, and repeated negative attention from anonymous or semi-anonymous users (Kavanagh et al., 2022; National Collegiate Athletic Association, 2026a). Recent institutional and scholarly reports have highlighted that online harassment is no longer a marginal issue but a recurrent stressor in highly visible sport environments (Kavanagh et al., 2022; National Collegiate Athletic Association, 2023, 2026a,b). This context raises a theoretically and practically important question: how is social media harassment associated with psychological distress among collegiate student-athletes, and what protective resources may weaken this association?

The present study focuses on collegiate student-athletes because they occupy a distinctive developmental and performance context. Unlike general university students, student-athletes must simultaneously manage academic requirements, intensive training schedules, competitive selection, team expectations, injury risk, and public evaluation. Unlike professional athletes, many collegiate student-athletes remain in a transitional stage of identity development and may have fewer professional resources for managing public scrutiny. Social media harassment can therefore enter an already demanding role structure. A hostile message after a poor performance may not be experienced merely as ordinary online conflict; it may be interpreted as an evaluation of athletic competence, team belonging, body image, personal identity, or institutional representation. In this sense, social media harassment represents a sport-relevant digital stressor rather than a simple extension of general social media use (Mao, 2025; Moore et al., 2025; Weber et al., 2023).

Although studies on cyberbullying and digital victimization have consistently shown that online harassment is associated with adverse psychological outcomes (Arif et al., 2024; Lee et al., 2026; Shannon et al., 2022), research specifically centered on student-athletes remains comparatively limited. Athlete mental health research has documented that sport participation does not protect athletes from psychological vulnerability and that competitive environments may contain distinctive stressors (Cosh et al., 2024; Kegelaers et al., 2024; Reardon et al., 2019). Yet the digital environment in which student-athletes now perform, communicate, and receive evaluation remains insufficiently integrated into sport psychology models. Existing work often treats social media as a broad communication space, a branding platform, or a measure of use intensity. The present study instead conceptualizes social media harassment as hostile, humiliating, aggressive, or threatening online content directed at the athlete role (Lin et al., 2025; Schmidt et al., 2022; Zhang et al., 2023). This distinction is essential because the psychological meaning of digital exposure depends not only on time spent online but also on the social-evaluative content encountered there.

A second limitation concerns mechanism. Prior studies frequently examine whether online harassment is associated with psychological distress, but less attention is paid to the psychological resources through which this association may occur. Psychological resilience is especially relevant in this regard. Resilience refers to the capacity to maintain or recover adaptive functioning under adversity (Connor and Davidson, 2003; Gao et al., 2026). In the context of social media harassment, resilience may help student-athletes reinterpret hostile comments, detach from unfair criticism, preserve self-worth, maintain performance focus, and recover from emotional strain. Conversely, repeated harassment may be associated with lower perceived coping capacity by increasing perceived uncontrollability and reducing confidence in handling public evaluation. In this study, resilience is conceptualized not as an immutable personality trait but as a perceived coping-related resource that can vary across stressful contexts. Therefore, resilience is a theoretically meaningful pathway through which digital harassment may be linked to psychological distress (Yu et al., 2024).

A third limitation concerns the complementary direct and buffering roles of perceived social support. Main-effect models propose that believing support is available is generally associated with better psychological functioning, including stronger coping resources and lower distress, regardless of whether a specific stressor is present. The stress-buffering model makes an additional claim: social support can change the strength of the association between a stressor and a psychological response by altering threat appraisal, expanding coping options, and reducing isolation (Cobb, 1976; Cohen and Wills, 1985; Dong et al., 2024; Luo et al., 2025). These roles are not mutually exclusive. For student-athletes, support from teammates, coaches, family members, friends, athletic staff, and campus mental health professionals may be directly associated with greater resilience and lower distress while also weakening the negative association between harassment and resilience. Support may also be negatively associated with reported harassment exposure because supportive networks can facilitate earlier reporting, privacy management, and disengagement from hostile interactions; however, the direction of this association cannot be established with cross-sectional data. The present study therefore retains the main effects of perceived support, reports its association with harassment and distress, and treats first-stage moderation as the focal buffering test.

The present study addresses these gaps by developing a moderated mediation model linking social media harassment, psychological resilience, perceived social support, and psychological distress among collegiate student-athletes. Specifically, the study asks whether social media harassment is positively associated with psychological distress, whether psychological resilience statistically mediates this association, whether perceived social support has direct associations with resilience and distress, and whether support moderates the first stage of the mediation pathway. To improve contextual breadth, the empirical sample includes collegiate student-athletes from university sport programs in China, the Republic of Korea, and the United States. The model positions harassment as a digital stressor, resilience as an individual coping-related resource, social support as a relational protective resource, and psychological distress as the focal mental health outcome. Because the data are cross-sectional, the model is interpreted as a theoretically grounded conditional association model rather than as definitive evidence of temporal causality.

The study makes three contributions. First, it extends athlete mental health research by integrating sport-relevant social media harassment into the analysis of collegiate student-athlete psychological distress. Second, it identifies psychological resilience as a statistically supported explanatory pathway through which hostile digital experiences are associated with distress. Third, it distinguishes the direct protective role of perceived social support from its stress-buffering role and situates both functions within the multi-source support ecology of student-athletes. Rather than viewing social media harassment only as an online conduct issue or a platform-management problem, this study treats it as a psychologically meaningful digital stressor that warrants coordinated attention from athletic departments, coaches, peers, families, and mental health services.

## 2 Related work

### 2.1 Social media harassment as a digital stressor in collegiate sport

Cyberbullying and online harassment research has shown that hostile digital interactions can produce emotional strain because they are persistent, rapidly disseminated, difficult to escape, and often occur in semi-public settings (Arif et al., 2024; Lee et al., 2026; Shannon et al., 2022). These features are particularly relevant to collegiate sport. Student-athletes may be evaluated publicly after games, identified easily through team rosters or social media accounts, and targeted by users who connect sport performance with personal blame. Drawing on research and guidance concerning athlete digital abuse and online harassment, social media harassment in this study refers to repeated or salient exposure to hostile, humiliating, aggressive, threatening, or demeaning online messages directed at a student-athlete's performance, identity, appearance, team affiliation, personal life, or athletic role (Kavanagh et al., 2022; National Collegiate Athletic Association, 2026b; Schmidt et al., 2022).

The construct differs from general social media use in three respects. First, general use intensity captures the amount of online activity, whereas harassment captures the hostile interpersonal content encountered online. Second, general social media use can include beneficial communication, team bonding, self-presentation, information seeking, and fan engagement, whereas harassment reflects a negative digital exposure. Third, sport-related harassment is often tied to visible performance and collective identity; hostile messages after a missed shot, a lost match, or a selection decision may be experienced as criticism of competence, team membership, or worthiness. A focused harassment construct therefore provides a clearer psychological explanation than broad measures of use intensity.

Social media harassment may be especially salient for collegiate student-athletes because sport performance is visible, emotionally charged, and often tied to institutional reputation. A controversial moment in competition can lead to intense online reaction, and the student-athlete may encounter that reaction repeatedly through comments, reposts, direct messages, screenshots, or team-related discussion spaces. For student-athletes, such reaction is not simply external commentary; it may become part of the psychological environment in which they train, study, recover, and interact with teammates. The digital environment thus extends the performance arena into everyday life and may transform competition-related evaluation into ongoing psychological pressure (Ding et al., 2023; Kavanagh et al., 2022; Lin et al., 2025; Schmidt et al., 2022).

### 2.2 Psychological distress among student-athletes

Psychological distress refers to non-specific negative emotional states that commonly include symptoms of anxiety, depressive mood, tension, and perceived inability to cope (Kessler et al., 2002; Wojujutari and Idemudia, 2024). In sport settings, distress can arise from performance pressure, role conflict, injury, academic demands, selection uncertainty, interpersonal conflict, and public scrutiny. Systematic reviews and consensus work have emphasized that athletes can experience mental health symptoms at meaningful levels and that sport environments may contain both protective and risk factors (Mao, 2025; Moore et al., 2025; National Collegiate Athletic Association, 2023; Reardon et al., 2019; Weber et al., 2023).

For collegiate student-athletes, psychological distress is particularly important because it can affect not only subjective well-being but also academic functioning, training engagement, recovery, social relationships, and help-seeking behavior (Cosh et al., 2024; Kegelaers et al., 2024). Distress may also be underreported when athletes perceive mental strain as a sign of weakness or fear that disclosure could affect their role within the team. These concerns make it important to examine distress through a framework that recognizes both risk exposure and protective resources.

The present study positions social media harassment as a digital extension of performance-related stress. When student-athletes are repeatedly exposed to hostile digital content, they may experience rumination, emotional exhaustion, self-doubt, and heightened vigilance. These reactions are consistent with the stress-and-coping framework, which argues that psychological outcomes depend on how individuals appraise and manage stressful demands (Lazarus and Folkman, 1984). Social media harassment may be appraised as uncontrollable, public, identity-relevant, and socially threatening, making it a plausible correlate of psychological distress.

Accordingly, the first hypothesis is proposed:

**H1:** Social media harassment is positively associated with psychological distress among collegiate student-athletes.

### 2.3 Psychological resilience as a mediating mechanism

Psychological resilience is commonly understood as the capacity to adapt positively, recover from adversity, and maintain functioning under stress (Connor and Davidson, 2003; Gao et al., 2026). In the present context, resilience is relevant because social media harassment may not be associated with distress only through exposure itself; it may also be associated with the psychological resources that student-athletes use to interpret and cope with negative experiences. A resilient athlete may view hostile comments as external noise, seek support, maintain performance focus, and recover emotionally. An athlete with lower perceived resilience may internalize harassment, experience stronger self-doubt, and have difficulty disengaging from negative feedback.

There are two reasons to treat resilience as a mediator rather than only as a background trait. First, resilience is not entirely static. Exposure to repeated stressors can be associated with lower perceived coping capacity, particularly when the stressor is uncontrollable or socially evaluative. Social media harassment may undermine perceived resilience by making student-athletes believe that criticism is unavoidable, public, and personally directed. Second, resilience is closely linked to psychological distress because it shapes the ability to regulate emotions, maintain perspective, and recover from negative events. Lower resilience may therefore be associated with greater vulnerability to distress (Yu et al., 2024; Zhang et al., 2023).

The proposed mediation pathway is consistent with stress-and-coping theory (Lazarus and Folkman, 1984). Social media harassment represents a digital stressor. Psychological resilience represents a coping-related resource. Psychological distress represents the emotional state associated with stress that exceeds perceived coping capacity. Under this framework, harassment is expected to be negatively associated with resilience, and lower resilience is expected to be associated with higher distress.

Accordingly, the following hypotheses are proposed:

**H2:** Social media harassment is negatively associated with psychological resilience among collegiate student-athletes.
**H3:** Psychological resilience is negatively associated with psychological distress among collegiate student-athletes.
**H4:** Psychological resilience mediates the association between social media harassment and psychological distress.

### 2.4 Perceived social support as a buffering resource

Perceived social support refers to the subjective belief that help, care, understanding, and assistance are available from meaningful social ties (Zimet et al., 1988). In the student-athlete context, perceived support should be understood as a multi-source resource. Support may come from teammates who normalize shared experiences, coaches who shape the climate for disclosure and reporting, family members and friends who provide emotional reassurance, athletic staff who offer practical guidance, and campus mental health professionals who provide confidential assistance (Graupensperger et al., 2020; National Collegiate Athletic Association, 2026b; Simons and Bird, 2023). Perceived support is particularly important because the belief that support is available may reduce threat appraisal even before support is actively used.

The stress-buffering model suggests that social support reduces the negative psychological impact of stress by altering appraisal, increasing coping options, and reducing feelings of isolation (Cobb, 1976; Cohen and Wills, 1985; Dong et al., 2024; Luo et al., 2025). In the context of social media harassment, perceived support may help student-athletes interpret hostile messages as unfair and external rather than as accurate self-evaluations. Supportive relationships may also provide spaces for emotional expression, advice, and practical action, such as documenting harassment, limiting exposure, reporting abuse, or seeking professional support.

Social support is therefore not conceptualized only as a moderator. Under the main-effect model, perceived access to caring and dependable relationships should be positively associated with resilience and negatively associated with distress (Cohen and Wills, 1985; Dong et al., 2024; Luo et al., 2025). Its association with harassment exposure is also relevant, but theoretically less directional: supportive athletes may receive help limiting or reporting abuse, whereas harassment itself may lead athletes to withdraw or reassess the availability of support. The present cross-sectional study reports this association without assigning a causal direction. The moderation test addresses a separate question—whether the harassment–resilience association differs depending on the level of perceived support after the corresponding main effects have been retained in the model.

The present study expects perceived social support to moderate the association between social media harassment and psychological resilience. When perceived support is high, harassment may show a weaker negative association with resilience because student-athletes can draw on relational resources to maintain confidence and recover from emotional strain. When perceived support is low, harassment may show a stronger negative association with resilience because negative digital experiences are more likely to be experienced as isolating and uncontrollable. This moderation focus also reflects the sport context: a supportive teammate or coach can change how an athlete appraises a hostile digital event before distress becomes more severe.

Accordingly, the following hypotheses are proposed:

**H5:** Perceived social support is positively associated with psychological resilience and negatively associated with psychological distress among collegiate student-athletes.
**H6:** Perceived social support moderates the association between social media harassment and psychological resilience, such that the negative association is weaker when perceived social support is higher.
**H7:** The indirect association between social media harassment and psychological distress through psychological resilience varies across levels of perceived social support.

### 2.5 Conceptual model

Figure 1 presents the conceptual model. Social media harassment is specified as positively associated with psychological distress directly and indirectly through lower psychological resilience. Perceived social support is specified to have direct associations with resilience and distress and to attenuate the negative association between harassment and resilience. The observed association between support and harassment is examined separately without imposing a directional causal path.

**Figure 1:**
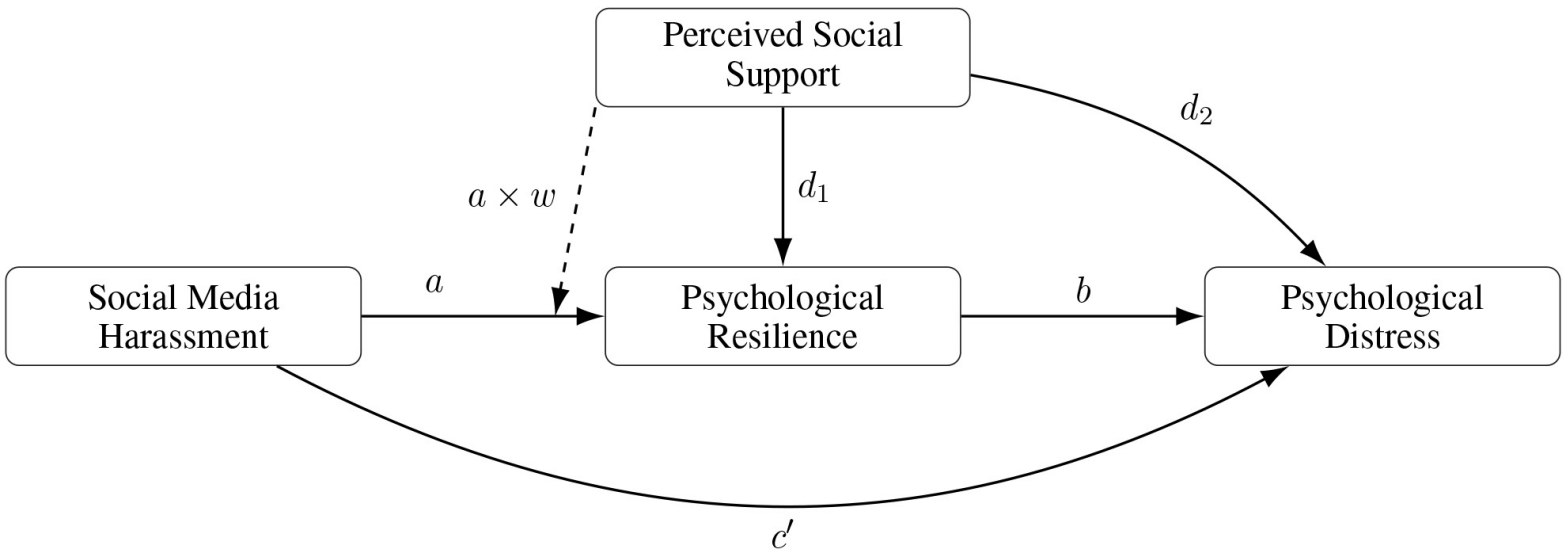
Conceptual model linking social media harassment, psychological resilience, perceived social support, and psychological distress. Perceived social support is modeled both as a direct protective correlate of resilience and distress and as a moderator of the first-stage harassment–resilience pathway. Its association with harassment is examined empirically but is not drawn as a directional path because the cross-sectional design cannot establish temporal ordering.

## 3 Methodology

### 3.1 Analytical design overview

This study adopted a cross-sectional survey design to examine theoretically grounded associations among social media harassment, psychological resilience, perceived social support, and psychological distress among collegiate student-athletes. The analytical design contained four connected stages. First, exposure to social media harassment was measured as a sport-relevant digital stressor surrounding athletic identity, competition, and public visibility. Second, psychological resilience was measured as a coping-related psychological resource. Third, perceived social support was measured as a multi-source relational resource that may be directly associated with resilience and distress and may attenuate the negative association between harassment and resilience. Fourth, psychological distress was measured as the focal mental health outcome.

The empirical strategy was designed to test mediation and moderation within a unified conditional process framework. The mediation component evaluated whether psychological resilience statistically explained part of the association between social media harassment and psychological distress. The moderation component evaluated whether perceived social support attenuated the association between harassment and resilience. The moderated mediation component evaluated whether the indirect association between harassment and distress through resilience differed across levels of perceived support. Because the study used cross-sectional data, all analyses were interpreted as conditional associations consistent with the theoretical model rather than as definitive evidence that harassment temporally caused later changes in resilience or distress.

### 3.2 Participants and procedure

Participants were collegiate student-athletes enrolled in university athletic programs in China, the Republic of Korea, and the United States. These three contexts were selected using two design criteria. First, they broadened institutional and cultural coverage by representing collegiate sport environments that differ in their organization and public visibility. Second, the six participating university sport programs could implement the same eligibility criteria, anonymous recruitment procedure, construct definitions, response scales, quality-screening rules, and language-adaptation protocol. This combination provided contextual breadth while preserving a common population definition: all participants were current students engaged in organized university training or competition. Although the three settings are not institutionally identical, student-athletes in each share the dual demands of academic study and competitive sport, regular evaluation by coaches and peers, and potential exposure to performance-related attention through social media. The study was not designed to produce nationally representative country comparisons. Instead, it tested whether the proposed individual-level association model was observable across three university-sport contexts under a common protocol.

Participants were recruited from six university sport programs: two universities in China, two universities in the Republic of Korea, and two universities in the United States. Within each site, athletic-program liaisons and student-athlete service offices distributed an anonymous survey link to eligible athletes. Coaches were permitted to forward the survey invitation but did not have access to individual responses. Pooling was based on identical eligibility criteria, construct definitions, response scales, quality-screening procedures, and translation/back-translation procedures. Country fixed effects were retained in the main models, and country-specific descriptive comparisons, country-by-harassment interaction tests, and leave-one-country-out sensitivity analyses were used to evaluate whether the pooled result was disproportionately driven by one setting.

Eligible participants were required to be at least 18 years old, currently registered as undergraduate or postgraduate students, and actively participating in organized university sport training or competition. The study targeted athletes from both individual and team sports to improve coverage across sport contexts. Competitive level was based on the highest level at which the participant had competed during the previous 12 months. Because labels differ across national systems, responses were harmonized into three analytical categories: university-level competition, subnational competition (regional, state, or provincial), and national-level competition. This harmonization was used for descriptive and covariate purposes and does not imply that the three sport systems are institutionally identical. A total of 476 eligible student-athletes were invited to participate, including 198 from China, 158 from the Republic of Korea, and 120 from the United States. Of these, 448 submitted questionnaires. After excluding responses with excessive missing data, invariant response patterns, failed attention checks, or extremely short completion times, the final analytical sample consisted of 426 collegiate student-athletes: 176 from China, 142 from the Republic of Korea, and 108 from the United States. The final sample included 232 (54.5%) male participants and 194 (45.5%) female participants. The mean age was 20.36 years (SD = 1.42).

The survey was administered through an online questionnaire in Chinese, Korean, or English, depending on the participant's university context and language preference. Participants first received information about the study purpose, voluntary participation, anonymity, data confidentiality, and the right to withdraw. After providing written informed consent electronically, they completed measures of social media harassment, psychological resilience, perceived social support, psychological distress, and demographic and sport-related covariates. To reduce common method bias, the questionnaire separated predictor, mediator, moderator, and outcome measures into different sections, used neutral item wording, varied response formats across constructs, and assured participants that there were no right or wrong answers. Participants were also informed that some questions concerned negative online experiences and emotional distress. They could skip any question or discontinue participation at any time, and information about campus counseling and psychological support services was provided at the end of the survey.

Sample adequacy was evaluated before the main analysis. An a priori power analysis for multiple regression with small-to-medium effects, alpha set at 0.05, and statistical power set at 0.80 indicated that the final sample size exceeded the minimum requirement for detecting the focal regression and interaction terms (Faul et al., 2009). The sample size was also considered adequate for confirmatory factor analysis and conditional process analysis because it provided sufficient observations relative to the number of estimated parameters. Because the sample was drawn from three country contexts, country/region was represented by dummy-coded fixed effects in the primary models. Country comparisons, country-by-harassment interaction tests, and leave-one-country-out specifications were additionally used to assess heterogeneity and the stability of the pooled estimates.

### 3.3 Measures

All measures were administered using self-report items. Where items were adapted for the student-athlete context, the wording was reviewed by two sport psychology scholars, two university athletic-program staff members, and one bilingual measurement researcher to ensure conceptual clarity and contextual relevance. For language adaptation, the questionnaire followed translation, back-translation, and discrepancy-resolution procedures commonly recommended for self-report measures (Beaton et al., 2000). The English item pool was translated into Chinese and Korean by bilingual researchers and then back-translated into English by independent translators. Discrepancies were discussed until semantic equivalence and contextual clarity were achieved. A small pilot check with 36 collegiate student-athletes from the three country contexts was conducted to evaluate item clarity, completion time, and sensitivity of wording. Pilot responses were not included in the final analytical sample.

#### 3.3.1 Social media harassment

Social media harassment was measured using an eight-item context-adapted scale assessing the frequency of hostile online experiences related to athletic participation (Kavanagh et al., 2022; Schmidt et al., 2022). The scale was designed to capture sport-relevant harassment rather than general social media use intensity. Items asked whether, during recent sport-related social media experiences, participants had received insulting comments about performance, humiliating posts or reposts, aggressive direct messages, performance-based blame after competition, unwanted personal attacks, threatening messages, appearance-related criticism, or repeated negative attention connected to their athlete role. Respondents rated items on a five-point scale ranging from 1 (never) to 5 (very often). Higher scores indicate greater exposure to social media harassment. Internal consistency for the scale was 0.91. The final item wording is reported in the supplementary item table to improve measurement transparency and replicability.

#### 3.3.2 Psychological resilience

Psychological resilience was measured using 10 items adapted from the Connor-Davidson resilience measurement tradition (Connor and Davidson, 2003). Items assessed perceived ability to recover from setbacks, remain focused under pressure, adapt to adversity, regulate emotional reactions, and maintain confidence after negative experiences. Respondents rated items on a five-point scale from 1 (strongly disagree) to 5 (strongly agree). Higher scores indicate greater psychological resilience. Internal consistency for the scale was 0.89.

#### 3.3.3 Perceived social support

Perceived social support was measured using 12 items adapted from the Multidimensional Scale of Perceived Social Support (Zimet et al., 1988). To reflect the collegiate sport context, the measure assessed perceived availability of emotional and practical support from family, friends, significant others, teammates, coaches, and athletic or campus support staff. Respondents rated items on a five-point scale from 1 (strongly disagree) to 5 (strongly agree). Higher scores indicate greater perceived social support. Internal consistency for the scale was 0.92.

#### 3.3.4 Psychological distress

Psychological distress was measured using 10 items adapted from the Kessler psychological distress framework (Kessler et al., 2002; Peixoto et al., 2021; Wojujutari and Idemudia, 2024). Items reflected non-specific distress symptoms, including nervousness, tension, worry, low mood, fatigue, and difficulty calming down during the recent period. Respondents rated items on a five-point scale ranging from 1 (none of the time) to 5 (all of the time). Higher scores indicate greater psychological distress. Internal consistency for the scale was 0.88.

### 3.4 Control variables

The analysis controlled for demographic, sport-related, and digital-exposure variables that may be associated with psychological distress. These included gender, age, academic status, country/region, sport type, competitive level, years of sport participation, weekly training hours, injury experience during the past year, and average daily social media use. Daily social media use was determined with a single open-response item: “During a typical day in the past 7 days, approximately how much total time did you spend using social media across all platforms?” Participants entered hours and minutes, which were converted to decimal hours per day and analyzed as a continuous covariate. This measure represented total exposure time rather than hostile content and was therefore conceptually distinct from the social media harassment scale. Including these covariates helped separate the focal associations from general differences in developmental stage, national collegiate sport context, sport participation intensity, public visibility, and digital exposure. Country/region was represented through dummy variables, with one category serving as the reference group in regression models.

### 3.5 Empirical strategy

The analysis proceeded in seven steps. First, descriptive statistics and bivariate correlations were calculated for all focal variables. Second, country differences were examined using one-way analyses of variance and gender differences were examined using independent-samples tests; effect sizes were reported to distinguish statistical from substantive differences. Third, reliability and validity were assessed using Cronbach's alpha, composite reliability, average variance extracted, confirmatory factor analysis, and discriminant-validity diagnostics. Fourth, hierarchical regression models tested the direct associations of social media harassment, psychological resilience, and perceived social support with psychological distress. A separate covariate-adjusted model examined the association between perceived support and reported harassment without assigning causal direction. Fifth, bootstrap mediation analysis estimated the indirect association between social media harassment and psychological distress through psychological resilience. Sixth, conditional process analysis tested whether perceived social support moderated the first-stage path and whether the indirect association varied across levels of perceived support (Hayes, 2022). Seventh, robustness checks examined whether the results remained stable across models with and without demographic and sport-related controls, with and without country/region fixed effects, using standardized variables, excluding low-quality responses, testing country- and gender-varying slopes, and estimating leave-one-country-out specifications.

The mediation model can be expressed as follows:


$$\begin{array}{l} M_{i}=\alpha_{0}+\alpha_{1}X_{i}+\alpha_{2}C_{i}+\varepsilon_{Mi}, \end{array}$$ (1)

$$\begin{array}{l} Y_{i}=\beta_{0}+\beta_{1}X_{i}+\beta_{2}M_{i}+\beta_{3}C_{i}+\varepsilon_{Yi}, \end{array}$$ (2)

where *X*_*i*_ denotes social media harassment, *M*_*i*_ denotes psychological resilience, *Y*_*i*_ denotes psychological distress, and *C*_*i*_ denotes the vector of control variables. The indirect association is calculated as α_1_β_2_.

The moderated mediation model adds perceived social support and the interaction term between social media harassment and perceived social support:


$$\begin{array}{l} M_{i}=\gamma_{0}+\gamma_{1}X_{i}+\gamma_{2}W_{i}+\gamma_{3}X_{i}W_{i}+\gamma_{4}C_{i}+\varepsilon_{Mi}, \end{array}$$ (3)

$$\begin{array}{l} Y_{i}=\delta_{0}+\delta_{1}X_{i}+\delta_{2}M_{i}+\delta_{3}W_{i}+\delta_{4}C_{i}+\varepsilon_{Yi}, \end{array}$$ (4)

where *W*_*i*_ denotes perceived social support. A significant coefficient for *X*_*i*_*W*_*i*_ indicates that perceived social support moderates the association between harassment and resilience. The conditional indirect association is expressed as (γ_1_+γ_3_*W*_*i*_)δ_2_.

### 3.6 Common method bias and robustness checks

Because the study used self-report survey data, several procedural and statistical remedies were adopted to reduce and evaluate common method bias (Podsakoff et al., 2003). Procedurally, the survey ensured anonymity, separated major constructs, used different response formats where appropriate, and emphasized that responses would not be shared with coaches or athletic administrators. Statistically, Harman's single-factor test and confirmatory factor comparisons were conducted to evaluate whether a single common factor accounted for the majority of covariance among items. A common latent factor model was also compared with the hypothesized four-factor model. Robustness checks included estimating models with and without demographic and sport-related controls, comparing specifications with and without country/region fixed effects, using standardized variables, testing alternative moderation positions, and examining whether results remained consistent after excluding participants with extremely short response times. These checks were used to evaluate the stability of the observed associations rather than to establish causal direction.

## 4 Data Collection and variable construction

### 4.1 Sample Characteristics

Table 1 summarizes the sample characteristics. The table reports country/region distribution, demographic information, sport participation characteristics, and social media use patterns. These variables are important because student-athletes may differ substantially in their exposure to online attention and in their vulnerability to psychological distress depending on national collegiate sport context, gender, sport type, competitive level, injury history, and training intensity.

**Table 1:** Sample characteristics of collegiate student-athletes.

Characteristic	Category	Value
Sample size	Total analytical sample	426
Country/region	China	176 (41.3%)
Country/region	Republic of Korea	142 (33.3%)
Country/region	United States	108 (25.4%)
Gender	Male	232 (54.5%)
Gender	Female	194 (45.5%)
Age	Mean (SD), years	20.36 (1.42)
Academic status	Undergraduate	379 (89.0%)
Academic status	Postgraduate	47 (11.0%)
Sport type	Team sport	267 (62.7%)
Sport type	Individual sport	159 (37.3%)
Competitive level	University	238 (55.9%)
Competitive level	Regional/state/provincial	146 (34.3%)
Competitive level	National	42 (9.9%)
Injury experience in past year	Yes	138 (32.4%)
Injury experience in past year	No	288 (67.6%)
Years of sport participation	Mean (SD), years	8.21 (3.37)
Weekly training load	Mean (SD), hours	15.84 (5.76)
Daily social media use	Mean (SD), hours	2.68 (1.14)

To make the competitive composition transparent across sites, Table 2 reports the harmonized competitive-level distribution within each country. The majority of participants in every country competed primarily at the university level, while each country also contributed athletes with subnational and national competition experience. The table documents the competitive composition of the pooled student-athlete sample and shows that participants were not restricted to a single competitiveness category. The descriptive distribution was not treated as evidence that the national sport systems were equivalent; pooling was instead supported by the common eligibility and measurement protocol and evaluated through the country-based sensitivity analyses described above.

**Table 2:** Competitive level by country/region.

Country/region	University	Regional/state/provincial	National	Total
China	88 (50.0%)	67 (38.1%)	21 (11.9%)	176
Republic of Korea	85 (59.9%)	45 (31.7%)	12 (8.5%)	142
United States	65 (60.2%)	34 (31.5%)	9 (8.3%)	108
Total	238 (55.9%)	146 (34.3%)	42 (9.9%)	426

Percentages in country rows are calculated within country. Competitive level refers to the highest level reported during the previous 12 months. Categories were harmonized for analysis and should not be interpreted as institutionally identical across countries.

### 4.2 Variable construction

For each construct, item scores were averaged after reverse coding where required. Social media harassment, psychological resilience, perceived social support, and psychological distress were standardized before interaction analysis to reduce multicollinearity and to facilitate interpretation. The interaction term was calculated as the product of standardized social media harassment and standardized perceived social support.

The dependent variable was psychological distress. The independent variable was social media harassment. The mediator was psychological resilience. The moderator was perceived social support. The primary control variables included demographic, country/region, sport-related, and digital-exposure covariates. Higher values on social media harassment represent stronger exposure to hostile online experiences, higher values on resilience represent stronger adaptive coping capacity, higher values on perceived support represent stronger perceived availability of supportive relationships, and higher values on psychological distress represent greater negative emotional symptoms.

### 4.3 Measurement model

A four-factor measurement model was estimated to verify the distinctiveness of the focal constructs. The model specified social media harassment, psychological resilience, perceived social support, and psychological distress as separate latent constructs. Model fit was evaluated using common fit indices, including CFI, TLI, RMSEA, and SRMR (Hu and Bentler, 1999). Convergent validity was examined through standardized factor loadings, composite reliability, and average variance extracted. Discriminant validity was assessed by comparing the square root of AVE with inter-construct correlations (Fornell and Larcker, 1981). The heterotrait-monotrait ratio was also inspected as an additional discriminant-validity diagnostic (Henseler et al., 2015). Measurement-model comparisons were used to examine whether the proposed four-factor structure fitted the data better than alternative models in which theoretically distinct constructs were combined.

## 5 Results

### 5.1 Preliminary comparisons by country and gender

Before estimating the pooled regression models, the focal variables were compared across country and gender groups. Table 3 reports group means, standard deviations, omnibus or two-group test statistics, and effect sizes. None of the country comparisons was statistically significant, and all country effect sizes were small (η^2^ = 0.004–0.005). Gender differences were also non-significant, with absolute standardized mean differences ranging from 0.09 to 0.16. These results do not establish population equivalence, but they indicate that the pooled model was not preceded by large observed mean differences in the focal variables. Country and gender were nevertheless retained as covariates, and slope heterogeneity was examined in sensitivity analyses.

**Table 3:** Focal-variable comparisons by country/region and gender.

	Country/region comparison						Gender comparison				
Variable	China	Republic of Korea	United States	*F*	*p*	η^2^	Male	Female	*t*	*p*	*d*
Social media harassment	2.36 (0.79)	2.48 (0.83)	2.41 (0.82)	0.86	0.423	0.004	2.47 (0.82)	2.34 (0.79)	1.69	0.092	0.16
Psychological resilience	3.68 (0.65)	3.57 (0.69)	3.60 (0.67)	1.15	0.318	0.005	3.66 (0.66)	3.57 (0.68)	1.35	0.178	0.13
Perceived social support	3.79 (0.69)	3.68 (0.73)	3.75 (0.71)	0.95	0.386	0.004	3.71 (0.72)	3.78 (0.70)	−0.96	0.339	−0.09
Psychological distress	2.24 (0.76)	2.35 (0.80)	2.28 (0.78)	0.79	0.455	0.004	2.24 (0.76)	2.35 (0.80)	−1.45	0.149	−0.14

Values for groups are mean (SD). Country comparisons used one-way analyses of variance; gender comparisons used two-sided independent-samples tests. Positive *d* values indicate higher male means. No test remained significant after Holm correction.

### 5.2 Descriptive statistics and correlation analysis

Table 4 reports means, standard deviations, reliability coefficients, and correlations among the focal variables. Social media harassment was positively correlated with psychological distress and negatively correlated with psychological resilience. Perceived social support was negatively correlated with both social media harassment and psychological distress and positively correlated with psychological resilience. Thus, the descriptive evidence already indicates that support was not operating only as a moderator; it also showed direct bivariate associations with the focal stressor, coping resource, and outcome.

**Table 4:** Descriptive statistics, reliabilities, and correlations among focal variables.

Variable	Mean	SD	Alpha	CR	AVE	1	2	3
1. Social media harassment	2.41	0.81	0.91	0.93	0.62	1		
2. Psychological resilience	3.62	0.67	0.89	0.90	0.56	−0.41^***^	1	
3. Perceived social support	3.74	0.71	0.92	0.93	0.64	−0.25^***^	0.43^***^	1
4. Psychological distress	2.29	0.78	0.88	0.89	0.55	0.48^***^	−0.52^***^	−0.36^***^

*N* = 426. CR, composite reliability; AVE, average variance extracted. ^***^p < 0.001.

The correlation matrix provides initial evidence for the theoretical model. Social media harassment showed a positive association with psychological distress, supporting the basic risk assumption of the study. Harassment was negatively associated with psychological resilience, indicating that hostile online experiences were linked to lower psychological coping resources. Psychological resilience was negatively associated with distress, supporting the proposed mediating mechanism. Perceived social support was positively associated with resilience and negatively associated with distress, consistent with a general protective main effect. Its negative correlation with harassment (*r* = −0.25, *p* < 0.001) indicates that athletes who perceived more support also reported less harassment exposure, although the cross-sectional design does not establish whether support reduced exposure, harassment altered support perceptions, or both were related to an unmeasured contextual factor.

Figure 2 provides a complementary visual summary of these descriptive and multivariable patterns. Figure 2a shows that the strongest bivariate association was the negative correlation between psychological resilience and psychological distress, followed by the positive association between social media harassment and distress. Figure 2b indicates that the focal variables were distributed across the response range rather than being concentrated at a single scale point. Figure 2c visualizes the standardized harassment and resilience coefficients from the mediation regression; the expanded model including the direct support coefficient is reported in Table 7.

**Figure 2:**
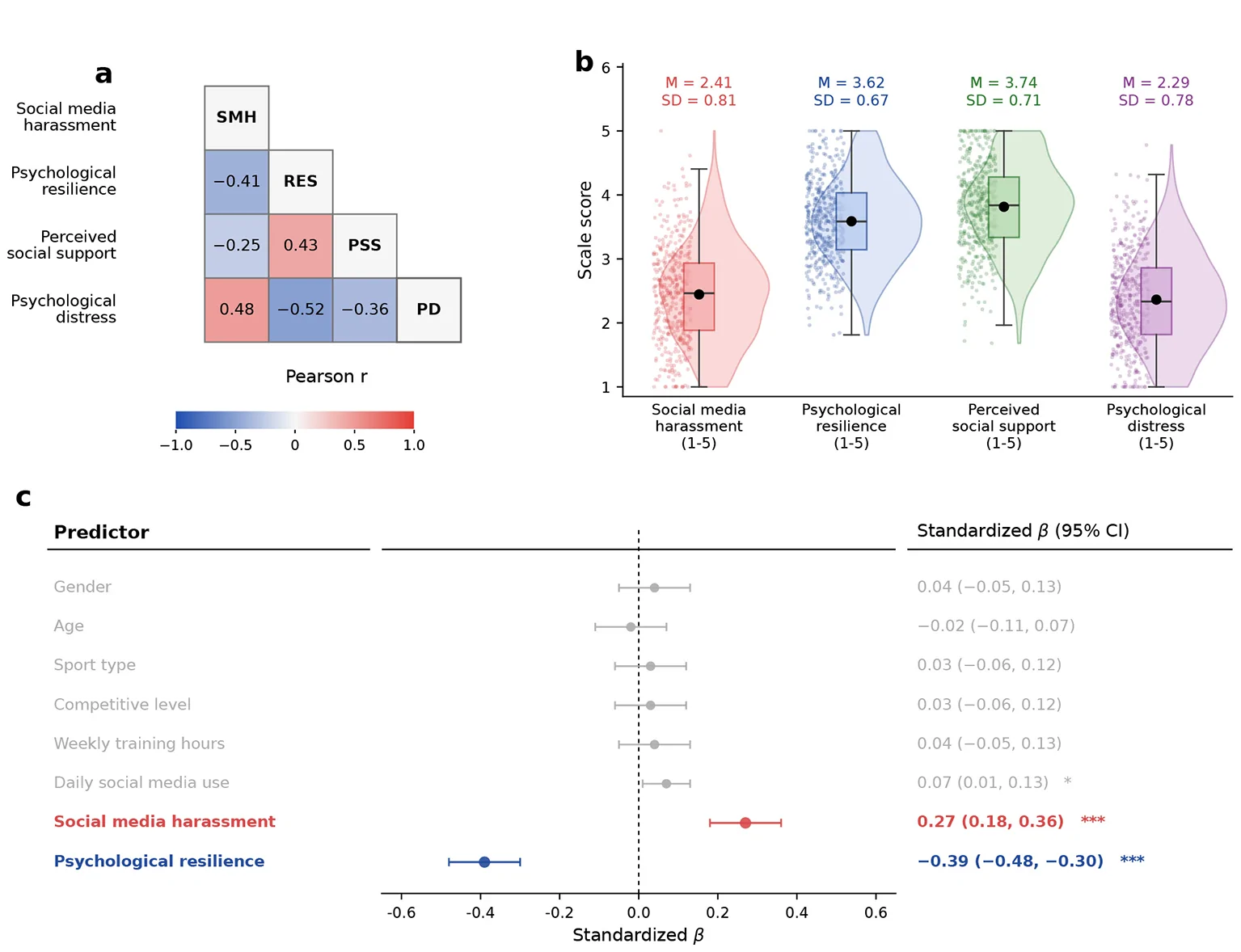
Descriptive distributions, bivariate correlations, and regression estimates for the focal variables. **(a)** Presents the lower-triangular Pearson correlation matrix among social media harassment, psychological resilience, perceived social support, and psychological distress. **(b)** Shows the distributions of the four focal variables with mean and standard-deviation annotations. **(c)** Reports standardized coefficients and 95% confidence intervals for social media harassment, psychological resilience, and selected covariates in the mediation regression predicting psychological distress. The direct perceived-support coefficient is reported in Table 7.

### 5.3 Reliability and validity

The four focal scales demonstrated acceptable internal consistency. Cronbach's alpha coefficients were 0.91 for social media harassment, 0.89 for psychological resilience, 0.92 for perceived social support, and 0.88 for psychological distress. Confirmatory factor analysis supported the four-factor structure, with fit indices of CFI = 0.956, TLI = 0.948, RMSEA = 0.046, and SRMR = 0.041. These results indicate that the focal constructs can be treated as empirically distinguishable dimensions in the subsequent hypothesis tests.

Table 5 summarizes the reliability and convergent-validity evidence for the measurement model. All factor-loading ranges were acceptable, composite reliability values exceeded 0.80, and AVE values exceeded the recommended threshold of 0.50. The maximum HTMT values remained below conservative cutoffs, supporting discriminant validity among the constructs.

**Table 5:** Reliability, convergent validity, and discriminant-validity diagnostics of the measurement model.

Construct	Items	Loading range	Cronbach's alpha	CR	AVE
Social media harassment	8	0.70–0.84	0.91	0.93	0.62
Psychological resilience	10	0.67–0.82	0.89	0.90	0.56
Perceived social support	12	0.68–0.86	0.92	0.93	0.64
Psychological distress	10	0.66–0.81	0.88	0.89	0.55

CR, composite reliability; AVE, average variance extracted.

Table 6 reports the measurement-model comparison. The hypothesized four-factor model fit the data better than alternative models in which theoretically distinct constructs were combined. The one-factor alternative model showed poor fit, and the first unrotated factor in Harman's single-factor test accounted for 28.4% of the variance. These results indicate that common method bias was unlikely to dominate the observed relationships.

**Table 6:** Confirmatory factor analysis model comparisons.

Model	χ^2^/df	CFI	TLI	RMSEA	SRMR
Four-factor model	1.78	0.956	0.948	0.046	0.041
Three-factor model: harassment and distress combined	3.02	0.885	0.871	0.069	0.073
Three-factor model: resilience and support combined	2.69	0.904	0.892	0.063	0.067
Two-factor model: risk and resource factors	3.63	0.849	0.833	0.079	0.088
One-factor model	6.33	0.621	0.589	0.132	0.119

CFI, comparative fit index; TLI, Tucker-Lewis index; RMSEA, root mean square error of approximation; SRMR, standardized root mean square residual.

### 5.4 Direct associations with psychological distress

Table 7 reports the hierarchical regression models predicting psychological distress. Model 1 includes the control variables. Model 2 adds social media harassment, which was positively associated with distress and supported H1. Model 3 adds psychological resilience; resilience was negatively associated with distress, supporting H3, and the harassment coefficient decreased, consistent with partial mediation. Model 4 adds perceived social support. Support retained a significant negative association with distress after harassment, resilience, country fixed effects, and the remaining covariates were controlled, supporting the distress component of H5. The increase in explained variance was modest but statistically meaningful, indicating that support contributed information beyond resilience rather than functioning only through the interaction term.

**Table 7:** Hierarchical regression models predicting psychological distress.

Predictor	Model 1		Model 2		Model 3		Model 4	
	β	SE	β	SE	β	SE	β	SE
Gender	0.08	0.05	0.05	0.04	0.04	0.04	0.03	0.04
Age	−0.04	0.04	−0.03	0.04	−0.02	0.04	−0.02	0.04
Academic status	0.05	0.04	0.03	0.04	0.02	0.04	0.02	0.04
Sport type	0.06	0.05	0.04	0.04	0.03	0.04	0.03	0.04
Competitive level	0.05	0.04	0.03	0.04	0.03	0.04	0.02	0.04
Years of sport participation	−0.03	0.04	−0.02	0.04	−0.02	0.04	−0.01	0.04
Weekly training hours	0.07	0.04	0.05	0.04	0.04	0.04	0.04	0.04
Injury experience	0.12^*^	0.05	0.09^*^	0.04	0.07	0.04	0.06	0.04
Daily social media use	0.15^**^	0.04	0.09^*^	0.04	0.07	0.04	0.06	0.04
Social media harassment			0.43^***^	0.04	0.27^***^	0.04	0.25^***^	0.04
Psychological resilience					−0.39^***^	0.04	−0.33^***^	0.04
Perceived social support							−0.17^***^	0.04
*R* ^2^	0.081		0.256		0.384		0.405	
Δ*R*^2^	–		0.175		0.128		0.021	
*F*	4.03^***^		14.31^***^		23.67^***^		23.44^***^	

Standardized coefficients are reported. All models controlled for country/region fixed effects. ^*^p < 0.05; ^**^p < 0.01; ^***^p < 0.001.

The support main effect complements rather than replaces the buffering test. In a separate covariate-adjusted model with social media harassment as the dependent variable, perceived social support was negatively associated with reported harassment (β = −0.22, SE = 0.05, *p* < 0.001). This adjusted association indicates that perceived support was related not only to psychological outcomes but also to reported harassment exposure. However, because support availability and harassment exposure were measured concurrently, the direction of this association cannot be determined. Consistent with Figure 2c, the final distress model indicates that harassment, resilience, and support each contributed in the theoretically expected direction.

### 5.5 Mediation analysis

The mediation analysis tested whether psychological resilience explains the association between social media harassment and psychological distress. Table 8 presents the path estimates. The path from social media harassment to psychological resilience corresponds to H2. The path from psychological resilience to psychological distress corresponds to H3. The indirect association corresponds to H4.

**Table 8:** Bootstrap mediation analysis of psychological resilience.

Path	Estimate	SE	95% CI lower	95% CI upper
Social media harassment → Psychological resilience (*a*)	−0.38^***^	0.04	−0.46	−0.30
Psychological resilience → Psychological distress (*b*)	−0.35^***^	0.04	−0.43	−0.27
Direct association (*c*′)	0.27^***^	0.04	0.19	0.35
Indirect association (*ab*)	0.133	0.025	0.088	0.185
Total association (*c*)	0.40^***^	0.04	0.32	0.48

Bootstrap confidence intervals were based on 5,000 resamples. ^***^p < 0.001.

The mediation hypothesis was supported because the bootstrap confidence interval for the indirect association did not include zero. Substantively, this result indicates that social media harassment was associated with higher psychological distress partly because it was associated with lower psychological resilience.

Figure 3 makes this mediation evidence easier to interpret by separating the indirect, direct, and total standardized associations. The indirect component was smaller than the remaining direct association, indicating partial rather than full mediation. The path-coefficient display also shows the expected directional pattern: harassment was negatively associated with resilience, resilience was negatively associated with distress, and harassment retained a positive direct association with distress.

**Figure 3:**
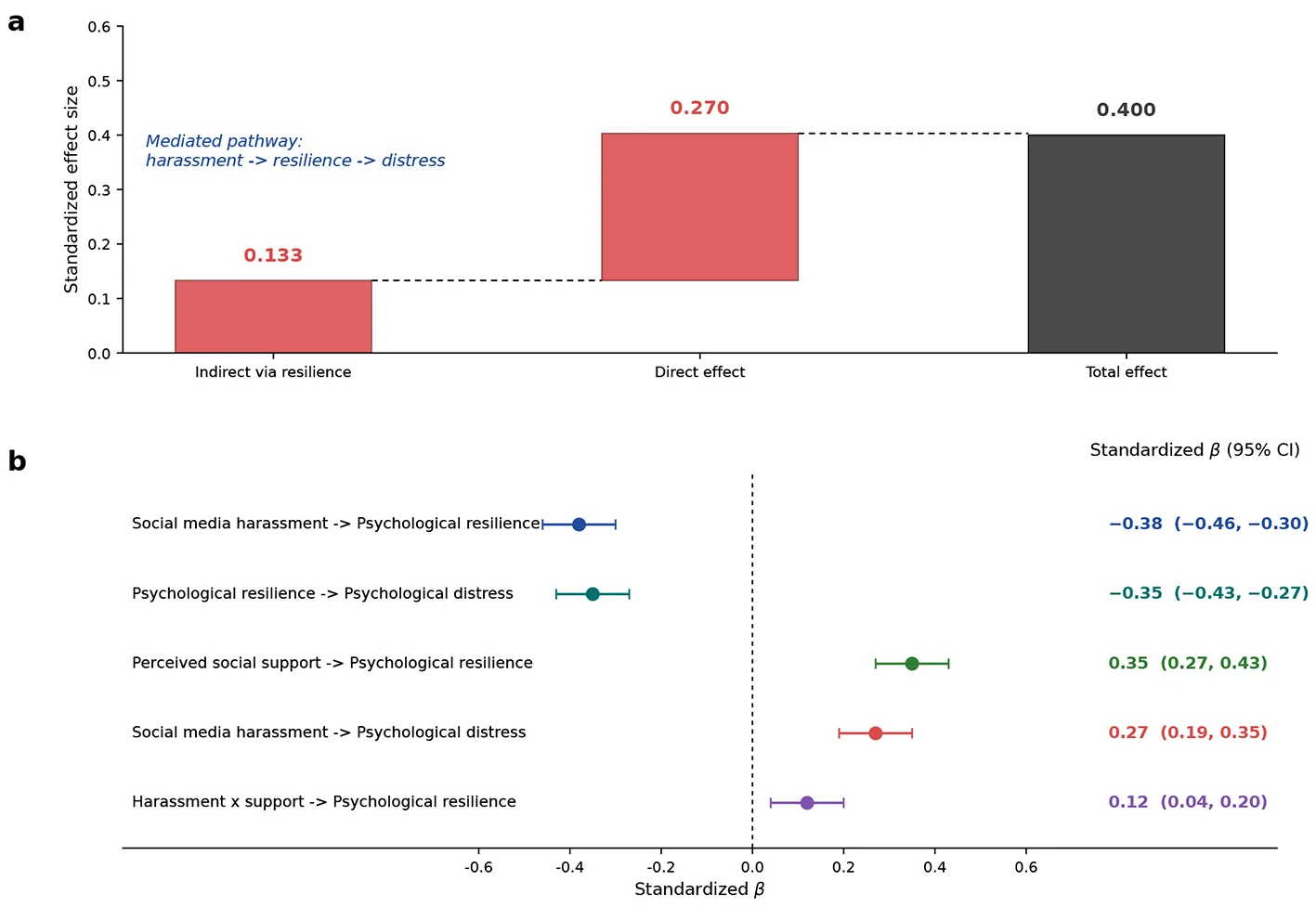
Effect decomposition and path estimates for the mediation model. **(a)** Decomposes the total standardized association between social media harassment and psychological distress into the indirect pathway through psychological resilience and the remaining direct association. **(b)** presents standardized path coefficients with 95% confidence intervals for the focal mediation and moderation-related paths.

### 5.6 Moderation analysis

The moderation analysis tested whether perceived social support attenuates the negative association between social media harassment and psychological resilience. Table 9 reports the regression model predicting psychological resilience. Perceived support showed a positive main effect on resilience, supporting the resilience component of H5, and the interaction between social media harassment and perceived social support was significant.

**Table 9:** Moderation model predicting psychological resilience.

Predictor	Estimate	SE	*t*	*p*
Social media harassment	−0.31	0.04	−7.75	< 0.001
Perceived social support	0.35	0.04	8.75	< 0.001
Harassment × perceived support	0.12	0.04	3.00	0.003
Control variables	Included	–	–	–
Country/region fixed effects	Included	–	–	–
*R* ^2^	0.354			
Δ*R*^2^ for interaction	0.014			

The significant interaction supports H6. Simple-slope analysis showed that the negative association between social media harassment and psychological resilience was stronger when perceived social support was low (*b* = −0.43, *p* < 0.001), moderate at the mean level of support (*b* = −0.31, *p* < 0.001), and weaker when perceived support was high (*b* = −0.19, *p* < 0.001). This pattern indicates that support functions as a protective relational resource in the face of hostile digital experiences.

Figure 4 illustrates this buffering pattern. When perceived social support was low, the fitted slope linking harassment to resilience was steeply negative, suggesting that hostile online experiences were more strongly associated with depleted coping resources. At the mean level of support, the slope remained negative but was less pronounced. When perceived support was high, the slope was still negative but substantially flatter, indicating that supportive relationships may help preserve resilience under social media harassment.

**Figure 4:**
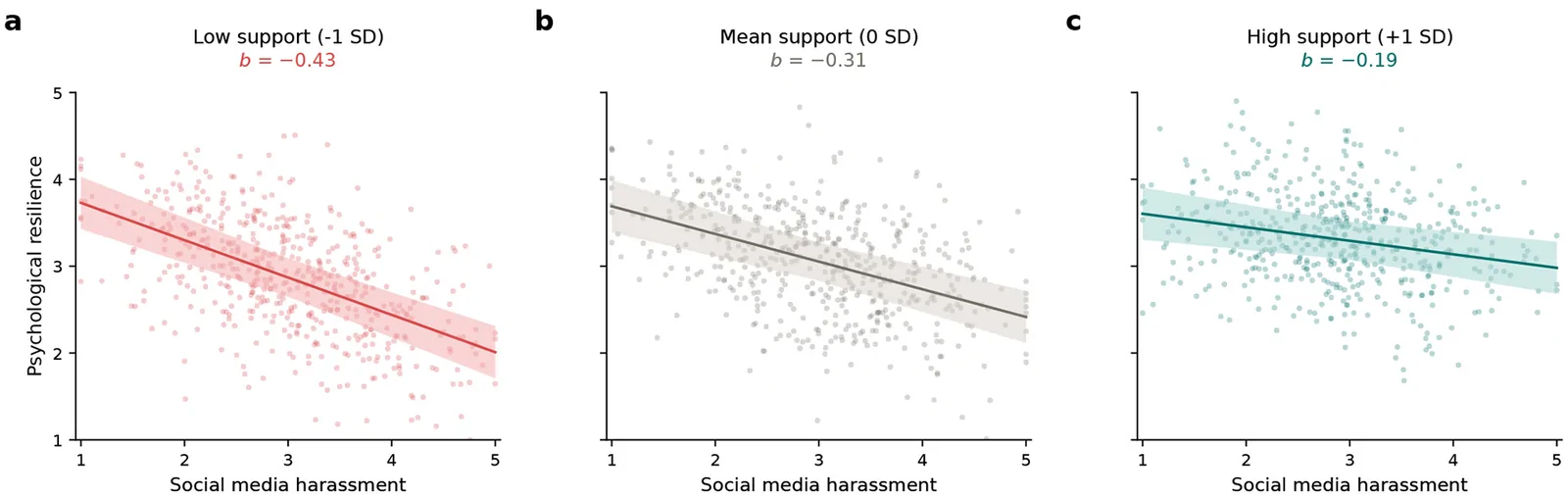
Simple-slope visualization of the moderation effect. **(a–c)** Show the association between social media harassment and psychological resilience at low, mean, and high levels of perceived social support, respectively. The weakening slope from low to high support indicates that perceived social support attenuates the negative association between harassment and resilience.

### 5.7 Moderated mediation analysis

The moderated mediation analysis examined whether the indirect association between social media harassment and psychological distress through psychological resilience differs across levels of perceived social support. Table 10 reports the conditional indirect associations at low, mean, and high levels of perceived social support.

**Table 10:** Conditional indirect associations across levels of perceived social support.

Level of perceived social support	Indirect association	BootSE	95% CI lower	95% CI upper
Low (−1 SD)	0.151	0.031	0.096	0.218
Mean	0.109	0.024	0.066	0.162
High (+1 SD)	0.067	0.022	0.030	0.115
Index of moderated mediation	−0.042	0.017	−0.078	−0.012

Bootstrap confidence intervals were based on 5,000 resamples.

H7 was supported because the confidence interval for the index of moderated mediation did not include zero. Social media harassment had a stronger indirect association with psychological distress through lower resilience when perceived support was low, whereas the indirect association was attenuated when perceived support was high.

Figure 5 further clarifies the conditional nature of the indirect association. The conditional indirect estimate decreased as perceived social support increased, and the confidence interval remained above zero across the plotted support range. The contrast between the low-support and high-support estimates is practically meaningful because it indicates that relational support does not merely add a direct protective resource; it also changes the extent to which harassment is associated with distress through reduced resilience.

**Figure 5:**
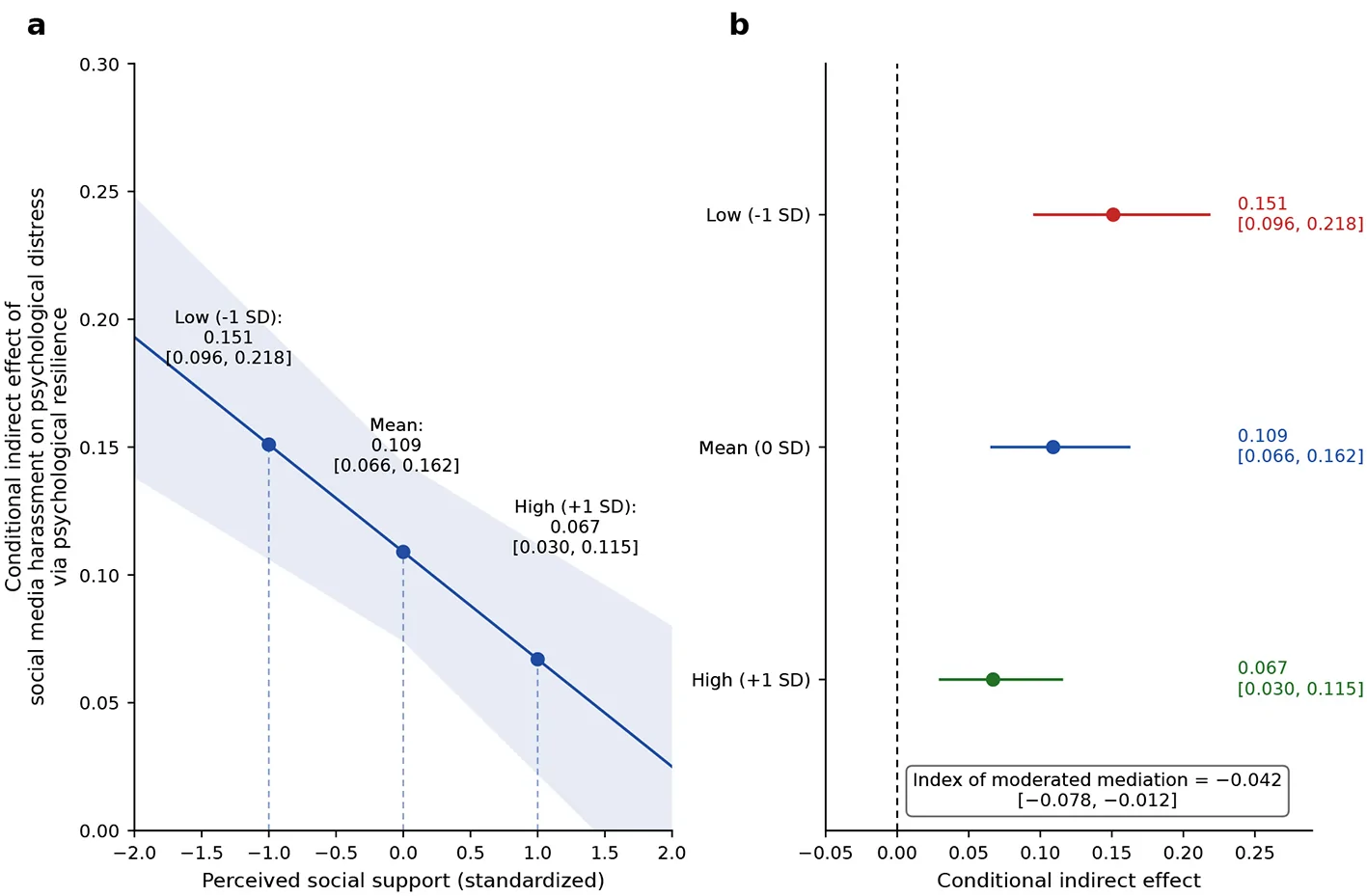
Conditional indirect association of social media harassment with psychological distress through psychological resilience across levels of perceived social support. **(a)** Displays the continuous change in the conditional indirect association across standardized perceived support. **(b)** summarizes the bootstrap estimates and 95% confidence intervals at low, mean, and high levels of perceived social support.

### 5.8 Robustness and sensitivity checks

Several robustness and sensitivity checks were conducted to examine whether the focal associations were dependent on a specific model specification. Table 11 summarizes the main results across alternative specifications. The primary model included demographic, sport-related, digital-exposure, and country/region fixed-effect controls. The pattern remained substantively consistent when country/region fixed effects were omitted, when demographic and sport-related controls were omitted, and when cases with extremely short completion times were excluded. Across these checks, social media harassment remained positively associated with psychological distress, psychological resilience remained a statistically supported explanatory pathway, and the harassment-by-support interaction remained positive in the model predicting resilience.

**Table 11:** Robustness checks for the moderated mediation model.

Specification	Direct association	Indirect association	Interaction	Index
Primary full model	0.27	0.133	0.12	−0.042
Without country/region fixed effects	0.28	0.131	0.11	−0.039
Without demographic and sport controls	0.29	0.139	0.11	−0.041
Excluding extremely fast responses	0.26	0.128	0.11	−0.040
Alternative standardized-variable specification	0.27	0.130	0.12	−0.043

The direct association refers to the standardized association between social media harassment and psychological distress after psychological resilience was included. The indirect association refers to the pathway through psychological resilience. The interaction refers to the harassment × perceived support term predicting psychological resilience. The index refers to the index of moderated mediation. The values are reported to show stability of direction and substantive interpretation across model specifications.

Additional subgroup-slope tests examined whether the focal associations varied by gender or country. The harassment-by-gender interaction was not significant when predicting resilience (β = 0.03, SE = 0.04, *p* = 0.481) or distress (β = 0.02, SE = 0.04, *p* = 0.612). Joint tests of the two harassment-by-country interaction terms were also non-significant for resilience (Δ*F* = 0.86, *p* = 0.424) and distress (Δ*F* = 0.91, *p* = 0.404). In leave-one-country-out analyses, the harassment-by-support coefficient ranged from 0.10 to 0.13 and the index of moderated mediation ranged from −0.038 to −0.046, with bootstrap intervals retaining the same interpretation. These results suggest that the pooled pattern was not attributable to a single country or to an observed gender-specific slope. They do not establish complete cross-cultural equivalence, however, and should be interpreted as sensitivity evidence rather than proof that the model is identical in all populations.

Taken together, the robustness checks indicate that the main pattern was not driven only by the cross-national composition of the sample or by a narrow set of control variables. At the same time, the checks do not remove the limitations of a cross-sectional self-report design. They should therefore be interpreted as evidence of model stability rather than evidence of temporal causality.

## 6 Discussion

### 6.1 Main findings

The present study examined social media harassment as a sport-relevant digital stressor associated with psychological distress among collegiate student-athletes. The proposed model integrated individual and relational protective resources by specifying psychological resilience as a mediating mechanism and perceived social support as both a direct protective correlate and a moderating condition. The findings indicate that social media harassment was positively associated with psychological distress, negatively associated with psychological resilience, and indirectly associated with distress through resilience. Perceived social support was directly associated with greater resilience and lower distress, was negatively associated with reported harassment, and attenuated the negative association between harassment and resilience. Thus, support showed complementary main-effect and buffering patterns rather than functioning only as an interaction variable.

These findings advance the understanding of student-athlete mental health by moving beyond the broad question of whether social media is harmful or beneficial in general. The evidence instead points to the importance of hostile digital interactions that are directly relevant to the athlete role. Social media harassment appears psychologically meaningful because it is linked not only to exposure to negative content but also to lower levels of a coping-related resource that may help athletes recover from adversity. The small country and gender mean differences, non-significant slope-interaction tests, and leave-one-country-out results indicate that the observed pattern was not concentrated in one measured subgroup, although the convenience sample does not support claims of national equivalence. These conclusions should be read as cross-sectional associations that are consistent with the theoretical model, not as proof of temporal or intervention effects.

### 6.2 Theoretical contributions

The first theoretical contribution is the conceptualization of social media harassment as a sport-relevant digital stressor. Prior cyberbullying research has established broad links between online harassment and negative mental health outcomes (Arif et al., 2024; Lee et al., 2026). The present study extends this work into collegiate sport by emphasizing the distinctive exposure patterns of student-athletes. Because sport performance is public, emotionally salient, and tied to team and institutional identity, harassment directed at athletes may have meanings that differ from ordinary online conflict. A hostile comment after a competition can be experienced not only as interpersonal negativity but also as an evaluation of competence, team membership, body image, or athletic identity.

The second contribution is the identification of psychological resilience as a mediating mechanism. Athlete mental health research often emphasizes risk factors such as injury, performance pressure, and stigma (Kegelaers et al., 2024; Reardon et al., 2019). This study adds that digital harassment may also be associated with psychological distress through lower perceived coping resources. By modeling resilience as the pathway between harassment and distress, the study provides a psychologically interpretable mechanism for future research. Importantly, resilience is not treated as a reason to expect athletes to tolerate harassment alone. Rather, it is treated as a coping-related resource that may be supported or undermined by the surrounding social and institutional environment.

The third contribution is the integration of the main-effect and stress-buffering models of perceived social support within a moderated mediation framework. The main-effect pattern was evident in the positive association of support with resilience and its negative association with distress after the other focal variables were considered. The buffering pattern was evident in the weaker negative harassment–resilience association at higher levels of support (Cobb, 1976; Cohen and Wills, 1985; Dong et al., 2024; Luo et al., 2025). The negative adjusted association between support and harassment further shows that support and exposure are empirically related, although the cross-sectional design precludes a directional interpretation. Distinguishing these roles helps bridge individual resilience theory and social support theory in the context of collegiate athlete well-being. It also clarifies that support in student-athlete populations is multi-source: teammates, coaches, family members, friends, athletic staff, and campus mental health professionals may all form part of the support ecology around online harassment (Graupensperger et al., 2020; Simons and Bird, 2023).

### 6.3 Practical implications

The findings suggest several cautious implications for universities, athletic departments, coaches, and mental health professionals. First, social media harassment may be considered a student-athlete welfare issue rather than only a reputational or communication problem. Universities and athletic departments may benefit from clear, confidential, and accessible reporting pathways linked to psychological support services. Student-athletes should know whom to contact when harassment occurs, how reports will be handled, and whether support can be accessed without affecting team status.

Second, athletic departments may consider preventive education that helps student-athletes recognize harassment, manage exposure, document abusive interactions, and seek support without fear of stigma (Kavanagh et al., 2022; National Collegiate Athletic Association, 2026a,b). Such education may be especially useful around high-visibility competitions, because championship monitoring has shown that higher-profile events can attract greater volumes of online abuse and threats (National Collegiate Athletic Association, 2024). Teams may also benefit from digital conduct guidelines that clarify how athletes, teammates, and staff can respond to online abuse in ways that protect privacy and psychological safety.

Third, resilience-oriented programs should be framed as potential support tools rather than as evidence that responsibility for coping belongs to the athlete. Existing sport psychology interventions provide concrete starting points: Mental Fortitude Training offers a structured framework for developing psychological resilience for sustained performance (Fletcher and Sarkar, 2016), and pressure-training interventions have been evaluated as a way to develop resilience-related capacities in competitive basketball settings (Kegelaers et al., 2021). These approaches were not designed specifically to prevent or treat social media harassment, so they should be regarded as transferable intervention models rather than as evidence of efficacy for online abuse. Programs adapted to the present context could combine coping skills, emotional regulation, cognitive reappraisal, attentional disengagement from hostile feedback, and recovery from public criticism with peer support, coach education, family communication, and accessible campus mental health services. This combined approach is consistent with the moderated mediation pattern, which suggests that resilience is embedded in relational and institutional conditions rather than functioning only as an individual attribute.

Fourth, coaches may occupy an important role in the support ecology around student-athletes, as coach–athlete relationship quality and social support have been associated with sport-related psychological well-being in collegiate athletes (Simons and Bird, 2023). Supportive coaching climates may help athletes perceive online harassment as external hostility rather than personal failure, although longitudinal and intervention evidence is needed to test this possibility directly. Coaches can also establish team norms that encourage reporting, peer support, and respectful digital behavior. Campus mental health services should also recognize that online harassment may be intertwined with performance pressure, identity development, help-seeking stigma, and belonging in sport communities (Cosh et al., 2024; Moore et al., 2025).

### 6.4 Limitations

Several limitations should be acknowledged. First, the cross-sectional design limits causal inference. Although the theoretical model specifies harassment, resilience, support, and distress in a directional framework, longitudinal data are needed to examine temporal ordering and changes over time. The mediation and moderated mediation findings should therefore be interpreted as conditional associations rather than as evidence of a confirmed causal sequence. Second, the use of self-report measures may introduce common method bias, although procedural and statistical remedies were used to reduce this concern. Third, social media harassment was measured through subjective reports. Future work could combine self-report data with digital diary methods, ecological momentary assessment, or platform-level exposure records where ethically appropriate.

Fourth, although the sample included collegiate student-athletes from China, the Republic of Korea, and the United States, it was not nationally representative of any country. The preliminary country comparisons, fixed-effect models, interaction tests, and leave-one-country-out analyses did not reveal large observed heterogeneity, but the country-specific subsamples had limited power to detect small differences. The questionnaire was administered in Chinese, Korean, and English, and translation, back-translation, expert review, and pilot checking were used to support semantic equivalence. Nevertheless, full configural, metric, and scalar measurement-invariance testing was not treated as a primary analysis. Country mean comparisons should therefore be interpreted descriptively rather than as definitive latent-mean comparisons, and future cross-national studies with larger probability-based samples should test measurement invariance directly. Fifth, gender mean differences and gender-varying slopes were small in the present sample, but this does not rule out heterogeneity by gender identity, sport type, competitive level, scholarship status, public visibility, or prior mental health history (Lee et al., 2026; Weber et al., 2023). Sixth, the social support construct was measured as perceived availability of support rather than observed support behavior. Future studies could distinguish emotional, informational, instrumental, peer, coach, family, and professional support in more detail.

### 6.5 Future research directions

Future research can extend this study in several ways. Longitudinal designs can test whether harassment predicts later changes in resilience and distress and whether resilience predicts recovery after digital harassment episodes. Experience sampling methods can capture short-term emotional reactions to online hostility and examine within-person fluctuations in threat appraisal, support seeking, and coping. Qualitative interviews can clarify how student-athletes interpret harassment, decide whether to disclose it, and evaluate the usefulness of support from coaches, teammates, family members, and university services. Future work can also distinguish different harassment types, such as performance-based blame, appearance-related comments, identity-based hostility, threatening messages, and repeated direct messaging. Finally, intervention studies can test whether resilience training, social support programs, coach education, and structured reporting systems are associated with lower distress among student-athletes exposed to online harassment (Ding et al., 2023; Yu et al., 2024; Zhang et al., 2023).

## 7 Conclusion

This study examined how social media harassment is associated with psychological distress among collegiate student-athletes and clarified the psychological and relational conditions underlying this association. By conceptualizing social media harassment as a sport-relevant digital stressor, the study moves beyond general discussions of social media use and focuses on hostile online experiences that are closely tied to athletic performance, public visibility, team identity, and emerging-adult development. In a multi-site cross-national convenience sample of collegiate student-athletes from China, the Republic of Korea, and the United States, higher reported exposure to social media harassment was associated with higher psychological distress.

The results further indicated that psychological resilience served as a statistically significant mediating mechanism. Social media harassment was negatively associated with psychological resilience, and lower resilience was associated with higher psychological distress. This pattern suggests that online harassment may become psychologically consequential not only because athletes encounter hostile comments or humiliating messages, but also because such experiences are associated with lower perceived coping capacity, emotional recovery, and confidence under pressure. These findings suggest that resilience should not be treated merely as a stable personal trait or as an individual burden placed on athletes. In the context of repeated digital stress, it may also reflect a coping-related resource that can be protected by the surrounding environment.

The study also highlighted the complementary roles of perceived social support. Support was directly associated with greater resilience and lower distress, and athletes reporting more support also reported less harassment exposure. In addition, the negative association between social media harassment and psychological resilience was weaker among student-athletes who perceived higher levels of support from family, friends, teammates, coaches, and other meaningful social ties. The conditional indirect association further indicated that the pathway from harassment to distress through lower resilience was stronger when perceived social support was low and weaker when perceived support was high. This pattern suggests the value of combining individual-level coping resources with relational and institutional support systems in future research and practice.

Overall, this study contributes to sport psychology and digital mental health research by identifying a moderated mediation pathway linking social media harassment, psychological resilience, perceived social support, and psychological distress among collegiate student-athletes. The findings suggest that universities and athletic departments should consider social media harassment as a student-athlete welfare issue rather than only as a communication or reputation-management problem. Because the study is cross-sectional, future longitudinal, diary-based, and intervention-oriented research is needed to examine temporal ordering and practical effectiveness. Future studies should examine how different forms of harassment develop over time, how student-athletes respond to digital stress in daily life, and whether structured resilience and social support interventions are associated with lower distress among athletes exposed to hostile online environments.

## Data Availability

The raw data supporting the conclusions of this article will be made available by the authors, without undue reservation.
