# Supplementary material for: Social media harassment and psychological distress among collegiate student-athletes: a moderated mediation model of psychological resilience and perceived social support
Source: Front Psychol. 2026 Oct 1;17:1885803. doi: 10.3389/fpsyg.2026.1885803 (PMC13668504; doi:10.3389/fpsyg.2026.1885803)
Supplement: Supplementary file 1 [file Data_Sheet_1.pdf]

Supplementary Material

SUPPLEMENTARY MATERIAL: SOCIAL MEDIA HARASSMENT ITEM POOL

To improve measurement transparency, the context-adapted social media harassment scale used the following eight item stems. Participants responded with reference to recent sport-related social media experiences on a five-point scale from 1 (never) to 5 (very often).

Table S1. Context-adapted social media harassment items.

Item	Stem
SMH1	I received insulting comments about my sport performance on social media.
SMH2	Other users posted or reposted humiliating content about me as an athlete.
SMH3	I received aggressive direct messages related to my team, competition, or athletic role.
SMH4	I was blamed online for a poor performance, loss, or team outcome.
SMH5	I experienced unwanted personal attacks connected to my identity as a student-athlete.
SMH6	I received threatening or intimidating messages through social media platforms.
SMH7	I received negative comments about my appearance, body, or personal life in connection with sport participation.
SMH8	I experienced repeated negative attention or hostile tagging related to my athlete role.
